# Comparing personalized brain-based and genetic risk scores for major depressive disorder in large population samples of adults and adolescents

**DOI:** 10.1192/j.eurpsy.2022.2301

**Published:** 2022-07-28

**Authors:** Gladi Thng, Xueyi Shen, Aleks Stolicyn, Mathew A. Harris, Mark J. Adams, Miruna C. Barbu, Alex S. F. Kwong, Sophia Frangou, Stephen M. Lawrie, Andrew M. McIntosh, Liana Romaniuk, Heather C. Whalley

**Affiliations:** 1 Division of Psychiatry, University of Edinburgh, Kennedy Tower, Royal Edinburgh Hospital, Morningside Park, Edinburgh, United Kingdom; 2 MRC Integrative Epidemiology Unit, University of Bristol, Bristol, United Kingdom; 3 Department of Population Health Sciences, Bristol Medical School, University of Bristol, Bristol, United Kingdom; 4 Djavad Mowafaghian Centre for Brain Health, University of British Columbia, Vancouver, British Columbia, Canada; 5 Department of Psychiatry, Icahn School of Medicine at Mount Sinai, New York, New York, USA

**Keywords:** Adolescents, genetics, imaging, major depressive disorder

## Abstract

**Background:**

Major depressive disorder (MDD) is a polygenic disorder associated with brain alterations but until recently, there have been no brain-based metrics to quantify individual-level variation in brain morphology. Here, we evaluated and compared the performance of a new brain-based ‘Regional Vulnerability Index’ (RVI) with polygenic risk scores (PRS), in the context of MDD. We assessed associations with syndromal MDD in an adult sample (*N* = 702, age = 59 ± 10) and with subclinical depressive symptoms in a longitudinal adolescent sample (baseline *N* = 3,825, age = 10 ± 1; 2-year follow-up *N* = 2,081, age = 12 ± 1).

**Methods:**

MDD-RVIs quantify the correlation of the individual’s corresponding brain metric with the expected pattern for MDD derived in an independent sample. Using the same methodology across samples, subject-specific MDD-PRS and six MDD-RVIs based on different brain modalities (subcortical volume, cortical thickness, cortical surface area, mean diffusivity, fractional anisotropy, and multimodal) were computed.

**Results:**

In adults, MDD-RVIs (based on white matter and multimodal measures) were more strongly associated with MDD (*β* = 0.099–0.281, P_FDR_ = 0.001–0.043) than MDD-PRS (*β* = 0.056–0.152, P_FDR_ = 0.140–0.140). In adolescents, depressive symptoms were associated with MDD-PRS at baseline and follow-up (*β* = 0.084–0.086, *p* = 1.38 × 10^−4^−4.77 × 10^−4^) but not with any MDD-RVIs (*β* < 0.05, *p* > 0.05).

**Conclusions:**

Our results potentially indicate the ability of brain-based risk scores to capture a broader range of risk exposures than genetic risk scores in adults and are also useful in helping us to understand the temporal origins of depression-related brain features. Longitudinal data, specific to the developmental period and on white matter measures, will be useful in informing risk for subsequent psychiatric illness.

## Introduction

Major depressive disorder (MDD) is a serious psychiatric disorder that significantly contributes to global disease burden [1, 2]. Large-scale neuroimaging approaches, such as the Enhancing Neuro Imaging Genetics through Meta-Analysis (ENIGMA) consortium, have greatly advanced the field by contributing robust findings on brain structural abnormalities associated with MDD [3–6]. However, these findings have limited clinical utility as they are based on group-level inferences and cannot be generalized across individuals that can have very different MDD profiles. This reflects the need for a more personalized approach to improve understanding of the biological origins of MDD. Recently, several novel brain-based metrics have been introduced to capture individual-level variation in brain morphology [7–11]. We focus here on the Regional Vulnerability Index (RVI) [9–11].

RVI is a personalized score that quantifies the degree of similarity (i.e., a correlation coefficient) between an individual’s brain pattern and the expected pattern of brain differences seen in a disorder, as determined by case–control effect sizes derived from large-scale meta-analyses (e.g., ENIGMA). Higher RVI indicates a stronger correlation and therefore, a higher vulnerability to the given disorder. An example would be that RVI based on white matter microstructural measures has been shown to differentiate schizophrenia patients from controls in clinical samples [9, 10] and also MDD cases from healthy individuals in a large epidemiological sample [11].

In this study, we derived six MDD-RVIs using different brain modalities (subcortical volume, cortical thickness, cortical surface area, mean diffusivity (MD), fractional anisotropy (FA), and multimodal) and examined their associations with syndromal MDD in a large adult sample. The comparison across modalities will help in identifying tissue types that are most implicated in MDD. Besides comparing MDD-RVIs, this study also evaluated the performance of MDD-RVIs relative to polygenic risk scores (PRS) for MDD. PRS is derived from the weighted sum of the number of risk alleles in an individual and has been shown to be associated with MDD across samples [12, 13]. We were therefore interested in using MDD-PRS as a benchmark to evaluate the validity of MDD-RVI in terms of effect sizes and sought to ascertain if both scores combined could account for more variation in disease risk than when used in isolation. Our hypothesis was that MDD-RVIs will show stronger associations with MDD than MDD-PRS, as the dynamic nature of brains structure across the lifespan may capture signals of additional risk factors beyond genetic risk that influence disease course.

Additionally, we repeated the analysis in a large adolescent sample, given that adolescence is the peak period for the onset of MDD [14–16]. However, adolescent MDD is often undiagnosed as symptoms are covert [17], resulting in continuity to adulthood [18]. We considered the investigation of MDD-RVI and MDD-PRS in the younger sample as exploratory, to determine the association between these personalized scores with cross-sectional and subsequent subclinical depressive symptoms. Specifically, MDD-RVIs for adolescents will be derived using effect sizes based on the adult meta-analyses in ENIGMA, to ensure sufficient statistical power and to see if brain features of vulnerable adolescents are similar to the adult MDD brain phenotypes. If brain-psychopathology associations are beginning to be established, this would have important implications for the identification of adolescents at increased risk of MDD. A continuous measure of depressive symptoms was used for the younger sample to better accommodate diagnostic uncertainty and capture the entire spectrum of severity [19].

## Methods

### Participants

#### GS-Imaging

Adult participants were from the deeply phenotyped imaging subsample of Generation Scotland: the Scottish Family Health Study (GS-Imaging). GS-Imaging received ethical approval from the NHS Tayside research ethics committee and all participants provided informed consent (reference 14/SS/0039). Information on the recruitment, assessment, and brain imaging procedures for this sample has been provided elsewhere [20]. The full GS-Imaging sample included 1,188 adults recruited across two sites in Scotland. The currently analysis comprised 702 unrelated and neurologically healthy individuals of European ancestry (age: 59 ± 10, 59% female). Further details are in Table 1 and in the Supplementary Materials.

**Table 1.** Demographic information for GS-Imaging, ABCD (baseline), and ABCD (2-year).

		Unit	GS-Imaging	ABCD (Baseline)	ABCD (2-year)
Demographics	Sample size	*N*	702	3,825	2,081
	Age	Years ± SD	59 ± 10	10 ± 1	12 ± 1
	Sex	% Females	59	47	44
MDD-RVI	Subcortical	*N* (healthy)	702 (524)	3,825 (3,218)	2,081 (1,732)
	Cortical	*N* (healthy)	702 (524)	3,825 (3,218)	2,081 (1,732)
	DTI	*N* (healthy)	686 (508)	3,630 (3,056)	2,032 (1,698)
MDD-PRS	MDD-PRS	*N*	702	3,825	2,081
Depressive phenotypes	Lifetime-MDD	*N* (cases/controls)	602 (223/379)	—	—
	TotalQIDS	*N*	702	—	—
		Mean ± SD	4.5 ± 3.7	—	—
	CBCL-DSM depressed	*N*	—	3,825	2,081
		Mean ± SD	—	1.3 ± 2.0	1.6 ± 2.3

*Note*: For the calculation of MDD-RVIs in our sample, subjects were deemed as healthy if they did not self-report any psychiatric diagnoses and were not taking antidepressants at the point of assessment.

Abbreviations: DTI, diffusion tensor imaging.

#### ABCD

Participants were from the Adolescent Brain and Cognitive Development (ABCD) study. The baseline sample comprised 11,875 youths recruited across 21 sites in the United States. Ethical approval was obtained from a central or local institutional review board [21]. Informed consent and assent were obtained from all parents and participants. Baseline and follow-up data from curated annual release 2.0 and 3.0, respectively, were obtained through the NDA database (https://nda.nih.gov/general-query.html?q=query=featured-datasets:Adolescent%20Brain%20Cognitive%20Development%20Study%20(ABCD); Federal-Wide Assurance: FWA00018101). The analysis sample comprised 3,825 unrelated and neurologically healthy individuals of European ancestry at baseline (age: 10 ± 1, 47% female) and a subset of 2,081 individuals (age: 12 ± 1, 44% female) at 2-year follow-up. Further details are in Table 1 and in the Supplementary Materials.

### Imaging measures

#### GS-Imaging

T1-weighted imaging and diffusion imaging data were obtained using the same protocol at either the Aberdeen study site (3T Philips Achieva TX-series MRI system Philips Healthcare, Best, the Netherlands) or the Dundee study site (Siemens 3T Prisma-FIT Siemens, Erlangen, Germany). T1 scans were processed using FreeSurfer 5.3.0 and the Desikan–Killiany atlas [22] was used for subcortical segmentation and cortical parcellation. FA and MD for white matter tracts were derived using TBSS toolkit within FSL following the ENIGMA DTI analysis protocol (http://enigma.ini.usc.edu/protocols/dti-protocols/). The John Hopkins University white matter atlas [23] was used to define white matter tracts. Full details on image acquisition and quality control measures are described in the Supplementary Materials and elsewhere [20, 24, 25].

#### ABCD

Minimally processed data for baseline and 2-year follow-up from the ABCD repository were used. Participants were scanned at 21 sites using 3T Siemens Prisma, General Electric 750 or Phillips scanner. Data acquisition and image processing methods were harmonized between sites and scanners [26, 27]. T1 scans were processed using FreeSurfer 5.3.0 and the Desikan–Killiany atlas was used for subcortical segmentation and cortical parcellation. Major white matter tracts were labeled using AtlasTrack [28]. Quality control was conducted following recommendations from the ABCD data team [26], and full details are in the Supplementary Materials.

### Regional Vulnerability Index

**Figure 1.**
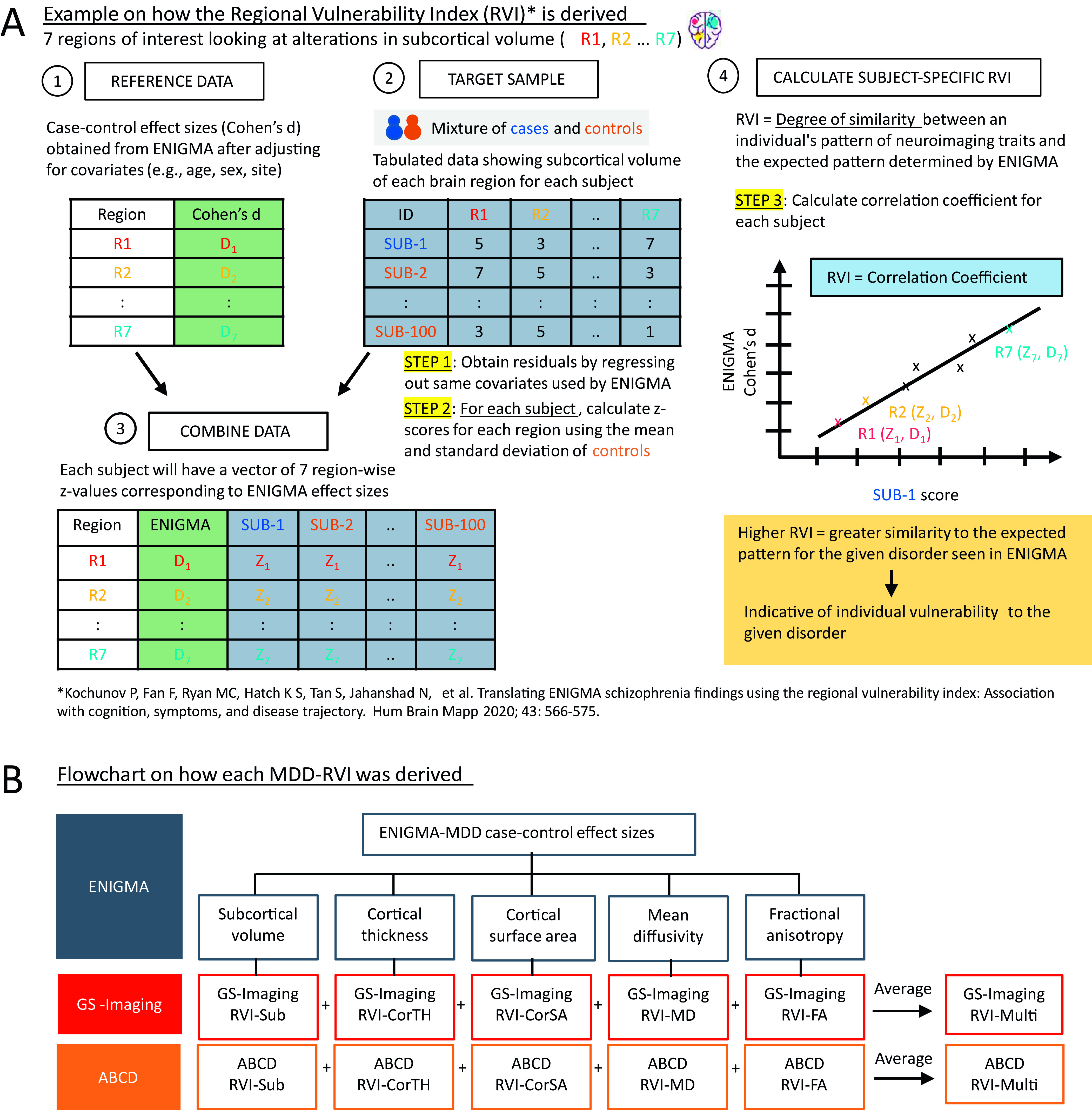
(A) A brief explanation on how MDD-RVIs for each modality are calculated, using alterations in subcortical volume as an example. The RVI method was developed by Kochunov et al. [10]. (B) The different types of MDD-RVIs that were derived for the GS-Imaging and ABCD samples, using MDD case–control effect sizes from ENIGMA meta-analyses.

MDD-related alterations in subcortical volume [3], cortical surface area and thickness [4], and tract-based MD and FA measures [5] were established by ENIGMA. Case–control effect sizes from these meta-analyses (Supplementary Tables S1–S4) were used as the reference data. MDD-RVIs were computed using the RVIpkg package (version 0.2.3) in R (https://cran.r-project.org/web/packages/RVIpkg/RVIpkg.pdf) and following the procedures specified by Kochunov et al. [10]. Briefly, for each brain region used in the calculation of an MDD-RVI type, the effects of covariates were first regressed out (see Supplementary Materials) and the residuals were *z*-normalized using the mean and standard deviation of healthy individuals in the sample. Subject-specific MDD-RVI was then calculated as a single Pearson’s correlation coefficient between the vector of the region-wise *z*-values and the corresponding regional effect sizes in the ENIGMA meta-analyses (see Figure 1A and the Supplementary Materials for details). The procedures were conducted separately for GS-Imaging and ABCD to define six MDD-RVIs for each subject: RVI-Sub for subcortical volumes, RVI-CorTH for cortical thickness, RVI-CorSA for cortical surface area, RVI-MD for mean diffusivity, RVI-FA for fractional anisotropy, and RVI-Multi, a multimodal index calculated as the average of the five RVIs (Figure 1B). GS-Imaging and ABCD were not part of the ENIGMA meta-analyses, thus ensuring no overlap between the discovery and testing samples.

### Polygenic risk scores

#### GS-Imaging

MDD-PRS was calculated for the full GS cohort, as detailed in Howard et al. [13]. Briefly, standard quality control measures and imputation using the Haplotype Reference Consortium dataset [29] were undertaken before deriving MDD-PRS using Plink v1.90b4 [30]. For the current analysis, only MDD-PRS for individuals within the GS-Imaging sample was included. Six *p*-value thresholds (pT_0.001, pT_0.01, pT_0.05, pT_0.1, pT_0.5, and pT_1) were considered, but only results for pT_0.1 are reported as the score at this threshold outperformed scores at different thresholds in this analysis in terms of effect sizes.

#### ABCD

The ABCD team conducted quality control on the genotyped data following the Ricopili pipeline [31] and then imputation using mixed ancestry and Eagle v2.4 phasing on the TOPMed imputation server using the full sample. Here, we only used imputed genetics data from unrelated individuals of European ancestry. In this subsample, we did post-imputation quality control by filtering out variants with INFO < 0.8 and minor allele frequency < 0.005. PRSice v2.1.11 [32] was used to calculate MDD-PRS using summary statistics by Howard et al. [13] using the clumping and thresholding method (clump-p = 1, clump-r2 = 0.25, clump-kb = 250 kb). A description of the phenotypes used in the summary statistics can be found in the Supplementary Materials. A linkage disequilibrium reference panel using the 1,000 Genomes central European population [33] was specified. MDD-PRS was obtained across the same *p*-value thresholds as in GS-Imaging, and pT_0.1 was chosen for subsequent analysis.

### Depressive phenotypes

#### GS-Imaging

Participants rated their symptoms over the past 7 days using a four-point Likert scale on the Quick Inventory of Depressive Symptomatology (QIDS) [34]. The total score (TotalQIDS) was used as a dimensional measure of current depressive symptoms. Categorical lifetime diagnosis of MDD (Lifetime-MDD) was determined using the Composite International Diagnostic Interview Short Form (CIDI-SF) [35] and the Structured Clinical Interview for DSM disorders (SCID) [36]. Both assessments were administered at overlapping intervals and were thus used to detect more MDD cases. Participants were defined as cases if they met the diagnostic criteria for CIDI or SCID, and controls if they did not meet the criteria for both.

#### ABCD

The primary caregiver completed the Child Behaviour Checklist (CBCL) [37] by rating the child’s behavior over the last 6 months using a three-point Likert scale [38]. Only caregiver-reported scores were used, as child-reported scores were not available. Other instruments completed by both caregiver and child were considered suboptimal, as binary measures may not be as effective in accommodating diagnostic uncertainty in adolescents. The total raw scores for the CBCL DSM-oriented depressive problems subscale (CBCL-DSM-Depressed) obtained at baseline and 2-year follow-up were used as dimensional measures of current depressive psychopathology.

### Statistical analysis

All statistical analyses were conducted using R (version 3.6.3). The associations between MDD-RVIs/MDD-PRS with depressive phenotypes were assessed separately in GS-Imaging and ABCD. For GS-Imaging, we used linear and logistic regression for models with TotalQIDS and Lifetime-MDD as outcomes, respectively. For ABCD, study site was modeled as a random effect in linear mixed models to account for the nested structure of the data. In GS-Imaging, site was included as a covariate as subjects were recruited only across two sites. In all models, covariates included age, age^2^, sex, site (for GS-Imaging), individual/parent education level and family income [10]. We additionally controlled for the top 15 genetic principal components and genotype plate number for analyses that included MDD-PRS as predictor. False discovery rate (FDR) correction was applied within each MDD-RVI type and within the selected MDD-PRS *p*-value threshold (pT_0.1). The change in *R* ^2^ was used to quantify the individual and combined explanatory power of MDD-RVI/MDD-PRS in terms of improvements in model fit relative to the null model (which only included covariates). The McFadden’s pseudo *R* ^2^ and marginal *R* ^2^ were used for logistic and linear mixed models, respectively. Analysis using the Akaike information criterion (AIC) was conducted to provide further evidence for the *R* ^2^ analysis. The above analytic approach was repeated for ABCD baseline and 2-year follow-up. We examined associations with CBCL-DSM-Depressed at each time point and with the change in symptoms between assessments. For the latter, we adopted a residualized change approach [39], whereby we residualized the 2-year CBCL-DSM-Depressed scores by regressing out baseline scores, sex, age difference, parent education, family income, and study site. Residuals were then regressed against baseline MDD-RVIs and MDD-PRS.

## Results

### Association between MDD-RVIs and depressive phenotypes

#### GS-Imaging

**Figure 2.**
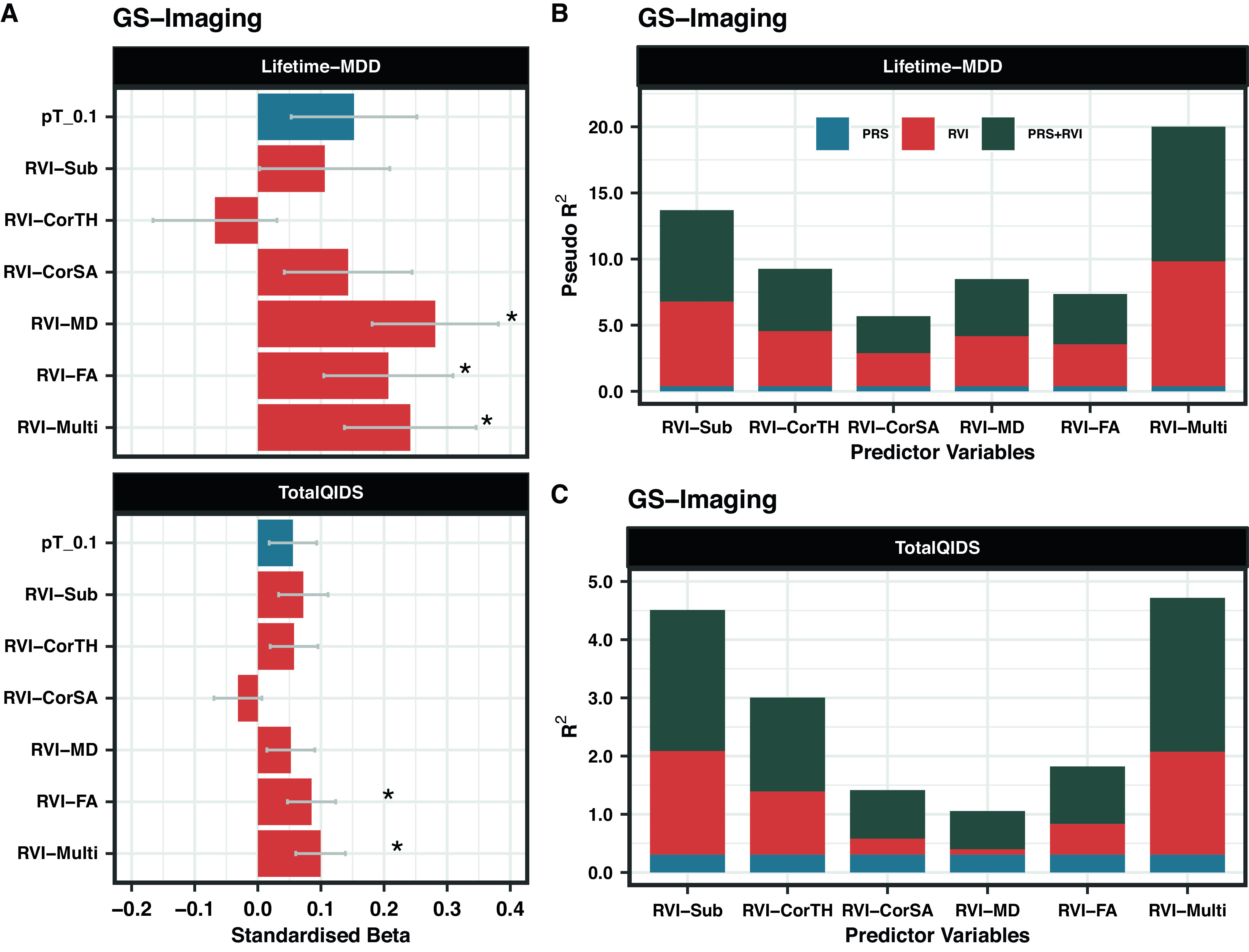
(A) Association between MDD-RVIs/MDD-PRS with Lifetime-MDD and TotalQIDS in GS-Imaging. The *x*-axis represents the standardized effect sizes and the *y*-axis represents the different MDD-RVIs and the MDD-PRS calculated at pT_0.1 threshold. (B) The change in McFadden Pseudo-*R* ^2^ (in %) contributed by each variable type (PRS, RVI, or PRS + RVI) when compared to a null model (i.e., covariates only) for Lifetime-MDD. (C) The change in *R* ^2^ (in %) contributed by each variable type (PRS, RVI, or PRS + RVI) when compared to a null model (i.e., covariates only) for TotalQIDS.

Lifetime-MDD was strongly associated with RVI-MD (*β* = 0.281, P_FDR_ = 0.001), RVI-FA (*β* = 0.206, P_FDR_ = 0.043), and RVI-Multi (*β* = 0.241, P_FDR_ = 0.021) (Figure 2A). Similar results were found for TotalQIDS for RVI-FA (*β* = 0.085, P_FDR_ = 0.043) and RVI-Multi (*β* = 0.099, P_FDR_ = 0.021), but not RVI-MD (*β* = 0.052, P_FDR_ = 0.169). Subcortical and cortical-based RVIs had no associations with either depressive phenotype (P_FDR_ > 0.05).

#### ABCD

**Figure 3.**
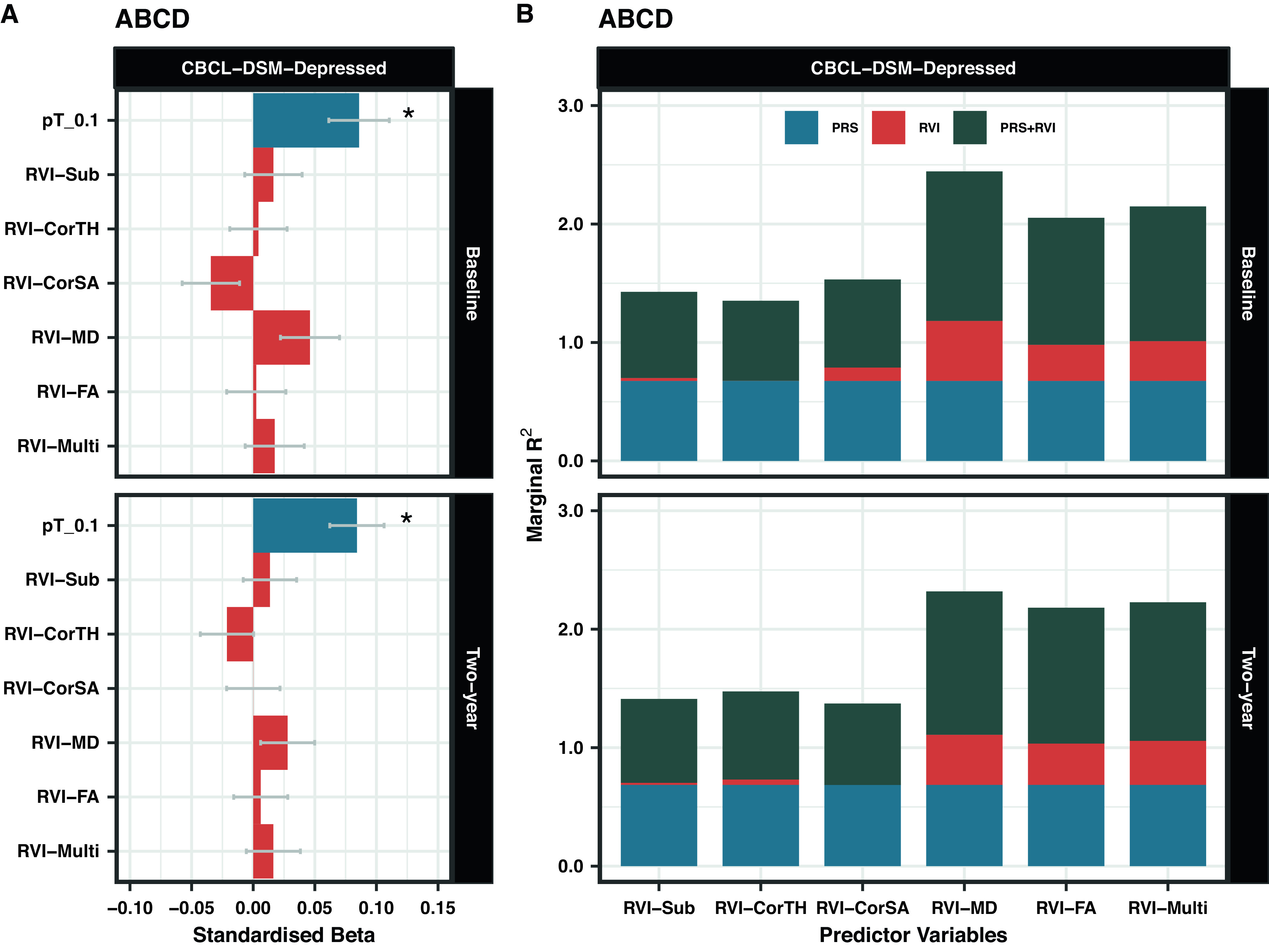
(A) Association between MDD-RVIs/MDD-PRS with CBCL-DSM-Depressed in ABCD at baseline and at 2-year follow-up. The *x*-axis represents the standardized effect sizes and the *y*-axis represents the different MDD-RVIs and the MDD-PRS calculated at pT_0.1 threshold. (B) The change in marginal *R* ^2^ (in %) contributed by each variable type (PRS, RVI, or PRS + RVI) when compared to a null model (i.e., covariates only) for CBCL-DSM-Depressed. The results at baseline and 2-year follow-up are reported.

CBCL-DSM-Depressed score was not associated with any MDD-RVIs either at baseline or at the 2-year follow-up (*β* < 0.05, *p* > 0.05, Figure 3A).

### Association between MDD-PRS and depressive phenotypes

#### GS-Imaging

MDD-PRS (at pT_0.1) was not associated with Lifetime-MDD (*β* = 0.152, P_FDR_ = 0.140) and TotalQIDS (*β* = 0.056, P_FDR_ = 0.140, Figure 2A). The results for other *p*-value thresholds of PRS are reported in Supplementary Figure S3. This finding may be related to reduced power in the imaging sample as analyses using the full unrelated GS sample (*N* = 6,946, including non-imaged participants) [13] revealed strong associations between MDD-PRS and lifetime MDD at all *p*-value thresholds (*β* = 0.182–0.204, *p* = 1.14 × 10^−7^−1.57×10^−6^, Supplementary Figure S4A).

#### ABCD

MDD-PRS was associated with CBCL-DSM-Depressed score at baseline (*β* = 0.086, *p* = 4.77×10^−4^) and at 2-year follow-up (*β* = 0.084, *p* = 1.38 × 10^−4^, Figure 3A). Results for other *p*-value thresholds are reported in Supplementary Figure S6.

### Comparing associations of MDD-RVI and MDD-PRS with depressive phenotypes

#### GS-Imaging

The effect sizes for the associations of RVI-MD, RVI-FA, and RVI-Multi with Lifetime-MDD (*β* = 0.206–0.281, P_FDR_ = 0.001–0.043) were higher than for MDD-PRS (at pT_0.1) (*β* = 0.152, P_FDR_ = 0.140, Figure 2A). The same was observed for RVI-Multi (*β* = 0.099, P_FDR_ = 0.021) and MDD-PRS (*β* = 0.056, P_FDR_ = 0.140, Figure 2A) in their associations with TotalQIDS.

#### ABCD

The effect sizes for the association between CBCL-DSM-Depressed for all RVIs (*β* < 0.05, *p* > 0.05) were consistently lower than those for MDD-PRS (at pT_0.1) at both time points (*β* = 0.084–0.086, *p* = 1.38×10^−4^−4.77×10^−4^, Figure 3A) and for other *p*-value thresholds (Supplementary Figure S6).

### Comparing change in R^2^ and AIC values of different model types using MDD-RVI and MDD-PRS as individual or combined predictors of depressive phenotypes

#### GS-Imaging

In models with Lifetime-MDD as the outcome, each MDD-RVI individually contributed to a greater change in *R* ^2^ (Pseudo-*R* ^2^ = 2.5–9.5%) than MDD-PRS (Pseudo-*R* ^2^ = 0%), with the strongest effect from RVI-Multi (Figure 2B). All MDD-RVIs contributed toward an improvement in model fit individually (compare M1 and M2) and additively with MDD-PRS (compare M3, M4, and M5) given the relative decrease in AIC values upon the addition of MDD-RVIs (Table 2). RVI-Multi contributed toward the largest decrease in AIC value in the full model relative to the null model. A similar pattern was observed with TotalQIDS as the outcome: all MDD-RVIs contributed toward an improvement in model fit individually and in combination with MDD-PRS (Table 2). Likewise, RVI-Multi contributed the largest change in *R* ^2^ individually (*R* ^2^ = 1.8%, Figure 2C) and had the largest additive contribution with MDD-PRS (Table 2), compared to other MDD-RVIs and MDD-PRS. Further calculations using delta AIC can be found in Supplementary Table S7.

**Table 2.** Absolute AIC values for each model type (M1–M5) when MDD-PRS and MDD-RVIs are used as predictors individually or in conjunction with each other

GS-imaging													
		Lifetime-MDD						TotalQIDS					
Model	Variables	RVI-Sub	RVI-CorTH	RVI-CorSA	RVI-MD	RVI-FA	RVI-Multi	RVI-Sub	RVI-CorTH	RVI-CorSA	RVI-MD	RVI-FA	RVI-Multi
M1	Covs (for RVI)	655.568						1,770.973					
M2	RVI+Covs (for RVI)	617.14	631.196	641.74	633.603	637.521	597.874	1,648.444	1,681.766	1,733.443	1,718.084	1,714.972	1,596.433
M3	Covs	672.293						1,792.363					
M4	PRS+Covs	671.93						1,792.09					
M5	PRS+RVI+Covs	633.582	647.215	659.033	649.654	652.779	613.38	1,667.764	1,701.706	1,753.004	1,737.658	1,735.256	1,614.54

ABCD													
		CBCL-DSM-depressed (baseline)						CBCL-DSM-depressed (2-year)					
Model	Variables	RVI-Sub	RVI-CorTH	RVI-CorSA	RVI-MD	RVI-FA	RVI-Multi	RVI-Sub	RVI-CorTH	RVI-CorSA	RVI-MD	RVI-FA	RVI-Multi
M1	Covs (for RVI)	4,970.509						5,791.486					
M2	RVI+Covs (for RVI)	4,972.006	4,972.475	4,970.297	4,713.946	4,717.684	4,717.155	5,793.091	5,792.503	5,793.486	5,628.348	5,629.907	5,629.424
M3	Covs	5,117.664						5,808.395					
M4	PRS+Covs	5,106.279						5,795.573					
M5	PRS+RVI+Covs	5,107.171	5,108.268	5,106.773	4,852.654	4,856.309	4,855.112	5,797.077	5,796.307	5,797.55	5,630.852	5,632.173	5,631.698

*Note*: AIC is a metric used to select the most parsimonious model that best explains the variance in the dependent variable. For example, the relative increase in AIC with the addition of new variable to the model would mean that the new predictor does not help to explain additional variance in the dependent variable. For model comparison, relative lower AIC values (typically at least two AIC units lower) are indicative of better model fit. The results for both GS-Imaging and ABCD are reported. *Covs: Covariates; “Covs (for RVI)” models do not include the 15 genetic principal components and genotype plate number that were included in the “Covs” models.

Abbreviations: AIC, Akaike information criterion.

#### ABCD

For both time points, among the MDD-RVIs, RVI-MD, RVI-FA, and RVI-Multi had the largest change in *R* ^2^ when added to the model individually (baseline *R* ^2^ = 0.3–0.5%, 2-year *R* ^2^ = 0.3–0.4%, Figure 3B). These three MDD-RVIs also contributed toward an improvement in model fit individually (compare M1 and M2) and in combination with MDD-PRS (compare M3, M4, and M5) at both time points, in contrast to the other MDD-RVIs (Table 2). MDD-PRS, however, accounted for a greater change in *R* ^2^ than each individual MDD-RVI at baseline (MDD-PRS *R* ^2^ = 0.7%, MDD-RVIs *R* ^2^ = 0–0.5%, Figure 3B) and at 2-year follow-up (MDD-PRS *R* ^2^ = 0.7%, MDD-RVIs *R* ^2^ = 0–0.4%, Figure 3B). MDD-PRS also contributed toward an improvement in model fit individually at both time points (Table 2).

### Association of MDD-RVIs and MDD-PRS with symptom change in ABCD

Mean CBCL-DSM-Depressed scores increased slightly from baseline (1.3 ± 2.0) to 2-year follow-up (1.6 ± 2.3). No MDD-RVIs nor MDD-PRS at any *p*-value thresholds was associated with symptom change (*β* < 0.05, *p* > 0.05, Supplementary Table S8).

## Discussion

This study examined associations between MDD-RVIs/MDD-PRS and depressive phenotypes in adults and further explored their utility in adolescents. White matter integrity-based MDD-RVIs (RVI-MD, RVI-FA) and RVI-Multi had the strongest associations among the MDD-RVIs in adults. These MDD-RVIs outperformed MDD-PRS in terms of effect sizes and contributed additively with MDD-PRS to model fit. This pattern did not generalize to early adolescence, where no significant associations were observed for any MDD-RVIs. MDD-PRS was, however, associated with adolescent depressive symptoms cross-sectionally but not with symptom change.

Our key findings on white matter integrity-based MDD-RVIs having the strongest associations with MDD phenotypes in adults are in line with results reported by previous studies looking at other adult cohorts using standard approaches [40, 41]. This suggests that reduced white matter integrity may be an important neurobiological feature of adult MDD and provides a rationale to look into finer details of the brain’s structural connectivity (e.g., connectomes) to elucidate the underlying mechanisms. Our findings also broadly replicate prior reports of RVI methods applied to UK Biobank, which reported significant associations between MDD and RVI-FA and RVI-Multi [11]. Notably, the FA and MD measures of individual tracts (rather than as a combined risk score) had very small effect sizes and no significant associations with symptom severity scores in the original ENIGMA meta-analysis [5] (see Supplementary Tables S5 and S6). This suggests that brain-based deviation of small effect sizes when considered collectively and across modalities can create individualized summary scores that are better measures of psychopathology than individual brain metrics in isolation.

Our results also support the role of MDD-PRS as an indicator of depressive phenotypes across the lifespan. The absence of any significant association in adults in this study is likely due to the lack of power given the relatively small sample size, and since MDD-PRS has been shown to be predictive in larger case–control studies [12, 13], including here in the larger GS sample. However, we considered it necessary to restrict the sample to individuals with both imaging and genetic data to ensure a fair comparison between MDD-RVIs and MDD-PRS. Given that MDD-PRS in adults also generalizes to adolescents (i.e., effect sizes for continuous depressive phenotypes were comparable in this study) [42], MDD-PRS may be useful as an early indicator of adolescent depression. The lack of association between MDD-PRS and the change in depressive symptoms in adolescents could be due to lower statistical power at the 2-year follow-up, or it could suggest that the changes are minimal due to the short time interval. Future work can consider following these young individuals over a longer time period for a more robust assessment of genetic contributions to MDD risk.

In evaluating the comparative performance of MDD-RVIs and MDD-PRS in adults, RVI-MD, RVI-FA, and RVI-Multi had higher associations with depressive phenotypes compared to MDD-PRS, especially for lifetime MDD. This may be because MDD-PRS and MDD-RVI operate on different timescales, with MDD-PRS being a snapshot one’s genetic susceptibility that is fixed since birth and MDD-RVI capturing the cumulative effects of non-genetic factors (e.g., environmental, biological) on the brain across the lifespan. The additive contribution to model fit by MDD-RVI and MDD-PRS, and the absence of a correlation between them (Supplementary Figure S2), indeed suggest that they each contribute toward unique variance. As such, it could be argued that MDD-RVI may be a useful additional measure of MDD risk in adults, since non-genetic risk factors can have an important influence on MDD risk, especially with the increase in exposure with age [43]. For example, depressed individuals who face stressful life events may have reductions in white matter integrity [44], which may play a mediating role between the environmental exposure and clinical outcome that is not captured to the same extent by genetic risk scores [45]. Given the significant role of environmental factors, future work could explore the effects of different environmental exposures on brain structural connectivity, such as through the use of methylation risk scores [46], which can also be combined with MDD-RVI and MDD-PRS to study their additive effects on MDD risk.

Notably, MDD-RVIs were not associated with depressive symptoms in the adolescent sample and underperformed compared to the MDD-PRS. This could be due to the use of effect sizes from the adult ENIGMA meta-analyses to derive the MDD-RVIs. The effect sizes were based on cases with established MDD and such estimates may not be directly relevant to cases with milder and less chronic depressive symptoms like in ABCD. Given that adults are likely to have more episodes and longer disease duration, the estimates may be more relevant within age group instead—for example, GS-Imaging cases with lifetime MDD, on average, have had three episodes of depression, each lasting for approximately 20 weeks (Supplementary Figure S5). In line with the above, adolescents may not yet demonstrate the same degree of detectable brain structural changes associated with depressive symptoms as adults, as psychopathology in adolescents can be transient and diagnostically uncertain [47, 48]. They might also have had lesser exposure to environmental/biological stressors of MDD, thereby cumulating lesser detrimental changes to the brain which are harder to detect. Given that adolescent brains are still undergoing significant developmental structural changes [49], the accuracy of the MDD-RVIs could have also been compromised due to the incorporation of variation attributed to neurodevelopment. As such, with the increase in sample sizes over the coming years, deriving adolescent MDD-RVIs using adolescent-specific effect sizes would be something important to look at in future, in order to identify brain-psychopathology associations, if any, that are unique to this age group. Nonetheless, it is interesting that adolescent RVI-MD, RVI-FA, and RVI-Multi (i.e., the best performing MDD-RVIs in the adult sample) had the highest proportion of variance explained among the other MDD-RVIs and were the only ones that contributed toward improved model fit individually and in combination with MDD-PRS. It is of interest to continue monitoring this longitudinal sample to see if the results become increasingly comparable to those seen in adults. We consider these findings critical for furthering the understanding of the role of neurodevelopment in shaping brain-psychopathology associations.

It is important to note that both MDD-RVI and MDD-PRS still only capture a small proportion of variance within the clinical phenotype and are not of clinical utility at present, either individually or in combination [50, 51]. Brain-based measures like MDD-RVI, however, are still potentially clinically useful, given our findings that effects of non-genetic risk factors can be captured by changes in brain structure. Its clinical potential may be enhanced in the near future, considering the steady increase in sample sizes of imaging studies (and genome wide association studies), which is indicative of a corresponding increase in power to detect more brain deviations (and genetic variants) that can hopefully contribute toward higher explained variance [52, 53]. Though not directly clinically useful yet, observations from such brain-based measures still inform our understanding of the biology of depression, which may aid in the development of future diagnostic biomarkers.

### Strengths and limitations

Strengths of this study include the large sample sizes, comparison of imaging and genetic predictors across different age groups, and the use of longitudinal data. Our findings should however be interpreted in the context of some limitations. Both GS-Imaging and ABCD are community-based samples consisting mainly of healthy individuals. We thus may not have captured the effects of moderate to severe MDD, but our results have the benefit of higher generalizability to the community. Our results may also not generalize to other ethnic groups as our sample was restricted to individuals of European ancestry. Future work can replicate this study in multi-ethnic cohorts when discovery data and analysis tools become more widely available. Additionally, we note that multiple factors, such as variations in protocols, can influence the reproducibility of imaging findings across sites. However, it was recently noted that neuroimaging findings, especially from large-scale studies, are generally consistent [54]. For RVI specifically, it has been shown that the method was effective when applied in different cohorts [9–11]. Along with the increase in adoption of standardized protocols (e.g., the use of ENIGMA’s DTI protocol in this study), there is more confidence in the replicability and applicability of results across sites [55–57].

## Conclusion

This study presents a comprehensive comparison of brain-based and genetic risk scores in terms of their association with MDD at different stages of the lifespan. MDD-RVIs, mainly those derived from white matter microstructural measures, had stronger associations with MDD and outperformed MDD-PRS in adults. While the contrary is true for adolescents, white matter-based MDD-RVIs, like in adults, contributed toward the highest proportion of variance explained among the MDD-RVIs. These findings are significant, as they inform our understanding of the temporal origins of depression-related brain features. They also highlight the importance of longitudinal studies for developing measures of risk for psychiatric illness, and the increasing influence of environmental exposures on brain structure and MDD risk across the lifespan.

## Data Availability

The data collected in the GS-Imaging study have been incorporated in the larger Generation Scotland dataset. Non-identifiable information from the Generation Scotland cohort is available to researchers in the United Kingdom and to international collaborators through application to the Generation Scotland Access Committee (access@generationscotland.org) and through the Edinburgh Data Vault (https://doi.org/10.7488/8f68f1ae-0329-4b73-b189-c7288ea844d7). Generation Scotland operates a managed data access process including an online application form, and proposals are reviewed by the Generation Scotland Access Committee. ABCD data repository grows and changes over time. The ABCD data used in this report came from the Annual Curated Release 2.0.1 and Release 3.0. Data can be accessed through registration with the ABCD study at https://nda.nih.gov/abcd.

## Supplementary material

For supplementary material accompanying this paper visit https://doi.org/10.1192/j.eurpsy.2022.2301. click here to view supplementary material
