# Supplementary material for: Comparing personalized brain-based and genetic risk scores for major depressive disorder in large population samples of adults and adolescents
Source: Eur Psychiatry. 2022 Jul 28;65(1):e44. doi: 10.1192/j.eurpsy.2022.2301 (PMC9393914; doi:10.1192/j.eurpsy.2022.2301)
Supplement: Supplementary file 1 [file S092493382202301Xsup001.docx]

**Comparing personalised brain-based and genetic risk scores for Major Depressive Disorder in large population samples of adults and adolescents**

Supplementary Materials

Thng *et al*.

**Supplementary Text**

1. **Exclusion criteria for GS-Imaging and ABCD**

**GS-Imaging**

For GS-Imaging, we excluded (a) individuals with neurological problems (brain tumour, hemotoma, stroke, epilepsy, haemorrhage, cerebral palsy and aneurysm, N=33), (b) related individuals, and (c) individuals with poor brain image quality (see Section 4 on Quality control measures for imaging data). As GS-Imaging is a family-based cohort, each participant within a family was assigned a random number and only one individual with the highest random number in each family was included.

**ABCD**

For ABCD, we excluded (a) individuals with neurological problems (N=114), (b) related individuals following the approach described for GS-Imaging, (3) individuals of non-European ancestry to match the MDD genome-wide association study discovery cohorts used to calculate PRS (see Section 2 on Selection of individuals of European ancestry in ABCD), and (4) individuals with poor image quality (see Section 4 on Quality control measures for imaging data).

1. **Selection of individuals of European ancestry in ABCD**

We restricted our sample to include only individuals of European ancestry to ensure the accuracy of polygenic risk scores, as summary statistics from genome wide association studies are typically based on European populations. Principal component analysis (PCA) was undertaken on ABCD genotyped data to generate the top 15 genetic principal components (PC). 4-means clustering was then performed on the top two PCs corresponding to White, Black, Hispanic and Asian. We created an intersection between the White clusters for PC1 and PC2 to generate a final White cluster. Individuals who self-reported as White, have parents that self-reported as White, and fall under the final White cluster were selected (N=4160). A PCA plot was done together with data from the 1000 Genomes Project Consortium [1] – European Population to confirm that subjects were selected accurately (Figure S1).

1. **Quality control steps for ABCD imputed genetics data**

The ABCD team conducted quality control (QC) on the genotyped data following the Ricopili pipeline [2] and then imputation using mixed ancestry and Eagle v2.4 phasing on the TOPMed imputation server (<https://topmedimpute.readthedocs.io/en/latest>) using the full sample. We filtered for INFO score<0.8 and then converted the genome build from hg38/GRCH38 to hg19/GRCh37 using LiftOver (<https://github.com/sritchie73/liftOverPlink>). As minor allele frequencies can differ between different ancestries, we further filtered for minor allele frequency <0.005 only after obtaining the European-only subsample.

1. **Quality control measures for imaging data**

**GS-Imaging**

Full image acquisition details can be found in Habota el al [3] T1 data were acquired from N=1,080 individuals. The FreeSurfer processed scans were visually inspected and minor errors were manually corrected. Errors included incorrect skull stripping, exclusion of grey or white matter in tissue segmentation maps, or incorrect brain parcellation into separate regions as detailed in Neilson et al [4]. Individuals were excluded when there was at least one major error that could not be corrected (e.g., in segmentation or cortical parcellation) or when there were multiple minor errors (N=12). Within those included, data was edited for N=424 individuals. Diffusion tensor imaging (DTI) data were acquired from N=1,058 individuals and processed using the TBSS toolkit. QC was performed following ENIGMA DTI protocols (<http://enigma.ini.usc.edu/protocols/dti-protocols/>), which included (1) correcting for eddy current-induced distortions and subject movement in the scanner, (2) skull stripping using BET at a threshold of 0.2, (3) using DTIFIT in order to compute diffusion tensor characteristics, and (4) visually checking the quality of FA images at this stage in order to exclude distorted images. In total, N=21 individuals were excluded due to errors during diffusion tensor fitting (N=14) and errors during registration/skeleton fitting (N=7). More details on QC measures can be found in Green et al [5] and Stolicyn et al [6]. The final analysis sample for GS-Imaging (N=702 after applying all exclusion criteria and merging with genetic and clinical data) included individuals with either T1 (N=702) or DTI (N=686) data, with N=680 having both.

**ABCD**

Full image acquisition details are detailed in Hagler et al [7]. The minimally processed data for the full sample were downloaded from the ABCD repository. Further QC following the QC recommendations by the ABCD team [7] were conducted for the unrelated European-only subsample. For morphometric measures, only individuals who had satisfactory T1 scans (field name: iqc_t1_ok_ser > 0) and passed FreeSurfer quality control (field name: fsqc_qc =1) were included (N=243 excluded). For white matter microstructural measures, only individuals who had satisfactory T1 scans, satisfactory diffusion MRI scans (field name: iqc_dmri_ok_ser > 0) and passed FreeSurfer QC were included (N=377 excluded). In addition to the standard quality control steps above, for baseline data only, average FA and MD values that were five standard deviations from mean were also removed to reduce skewness (N=18), as described in Shen et al [8]. The final analysis sample for ABCD baseline (N=3,825 after applying all exclusion criteria and merging with genetic and clinical data) included individuals with either T1 (N=3,825) or DTI (N=3,630) data, with N=3,630 having both. The QC process was repeated for the two-year follow-up assessment. In total, N=30 and N=79 individuals were excluded for morphometric measures and white matter microstructural measures, respectively at two-year follow-up. The final analysis sample for ABCD two-year follow-up (N=2,081 after applying all exclusion criteria and merging with genetic and clinical data) included individuals with either T1 (N=2,081) or DTI (N=2,032) data, with N=2,032 having both.

1. **Calculation of Regional Vulnerability Index (RVI)**

Using RVI-Sub as an example, the residuals for each of the 7 subcortical structures were first estimated by regressing out the effects of the same covariates used in the ENIGMA meta-analysis (refer to Table S1 to S4 for covariates used), and then z-score transformed using the mean and standard deviation of healthy individuals. In our sample, subjects were deemed as healthy if they did not self-report any psychiatric diagnoses and were not taking antidepressants at the point of assessment (Table 1 in main text). This procedure yielded a vector comprising 7 region-wise z-scores for each subject. Subject-specific RVI-Sub was then calculated as a single Pearson’s correlation coefficient between the vector of the 7 region-wise z-values and the corresponding regional effect sizes in the ENIGMA meta-analyses. This procedure was repeated for cortical thickness and surface area measures (using 33 cortical regions each), and for FA and MD measures (using 24 white matter tracts each), each yielding a single RVI. For ABCD, only 12 white matter tracts were used upon matching the tracts defined by AtlasTrack used in ABCD and the John Hopkins University atlas used in ENIGMA and GS-Imaging (Table S3). Measures from the left and right hemisphere were averaged. The RVI computation was implemented in the RVIpkg package version 0.2.3 in R (<https://cran.r-project.org/web/packages/RVIpkg/RVIpkg.pdf>).

1. **Phenotypes included in summary statistics used for PRS calculation**

Summary statistics were obtained from Howard et al [9]. Three different cohorts were included in the meta-analysis: UK Biobank, 23nMe and Psychiatric Genetics Consortium (PGC). Within UK Biobank, the broad definition of depression was used, where subjects were asked: ‘Have you ever seen a general practitioner for nerves, anxiety, tension or depression?’ or ‘Have you ever seen a psychiatrist for nerves, anxiety, tension or depression?’. Within 23nMe, phenotypic status was based on responses to web-based surveys, with individuals that self-reported as having received a clinical diagnosis or treatment for depression classified as cases. For PGC, cases were required to meet international consensus criteria (DSM-IV, ICD-9, or ICD-10) for a lifetime diagnosis of MDD established using structured diagnostic instruments from assessments by trained interviewers, clinician-administered checklists, or medical record review.

**Supplementary Figures**


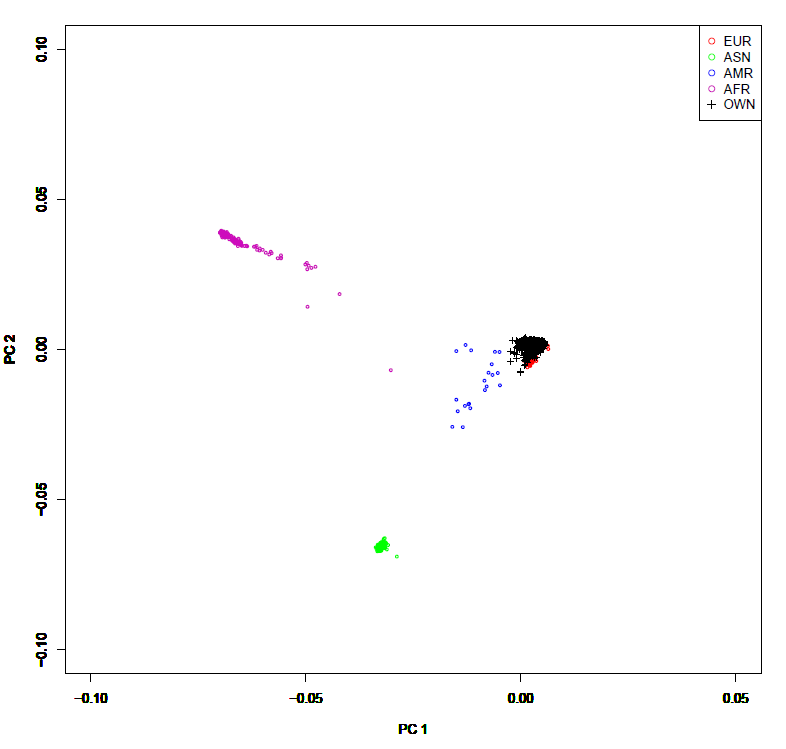


**Figure S1.** PCA plot using ABCD genotyped data and 1000 Genomes genetic data. The ABCD sample used in the current analyses were mostly of European ancestry, as indicated by the overlap with the 1000 Genomes European population. Figure legend: EUR – European, ASN – Asian, AMR – American, AFR – African, OWN – ABCD European-only sample.

**
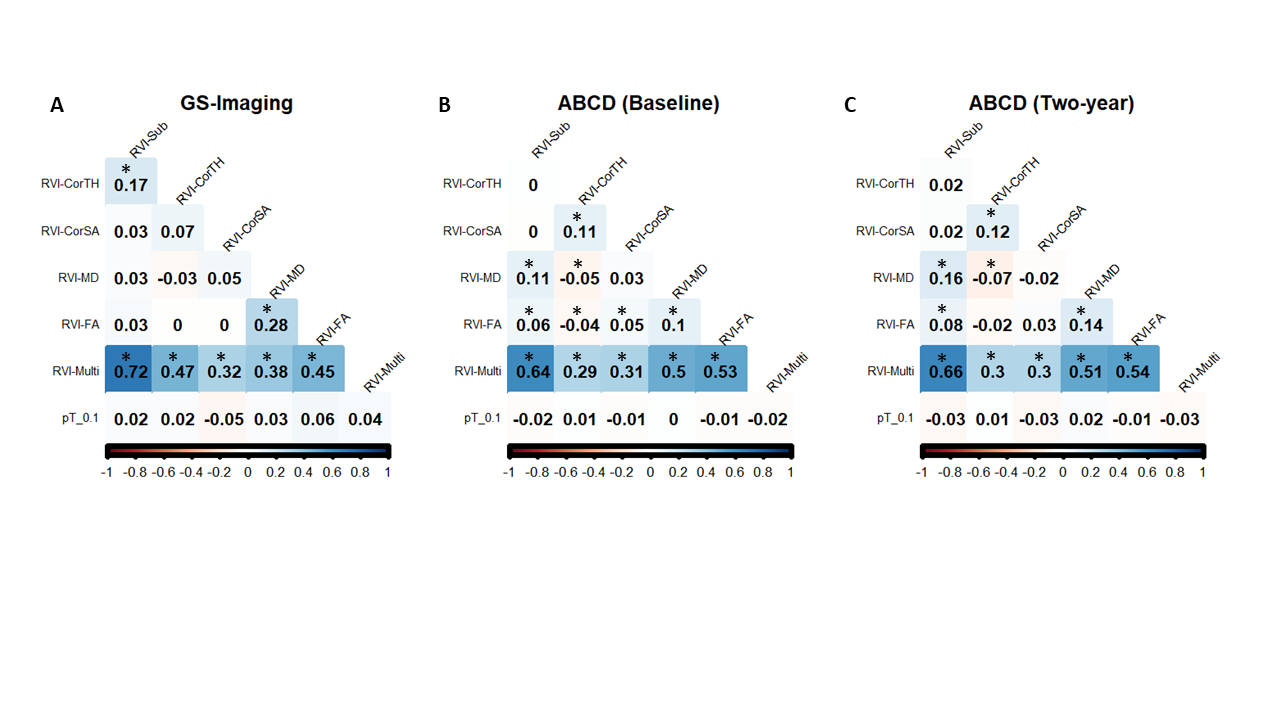
**

**Figure S2.** Correlation coefficients between the different MDD-RVIs and MDD-PRS at pT_0.1 for (A) GS-Imaging, (B) ABCD (Baseline), and (C) ABCD (Two-year). Boxes with “*” indicate that the correlation coefficient is significant at p<0.05.

**Supplementary figures for GS-Imaging**

**
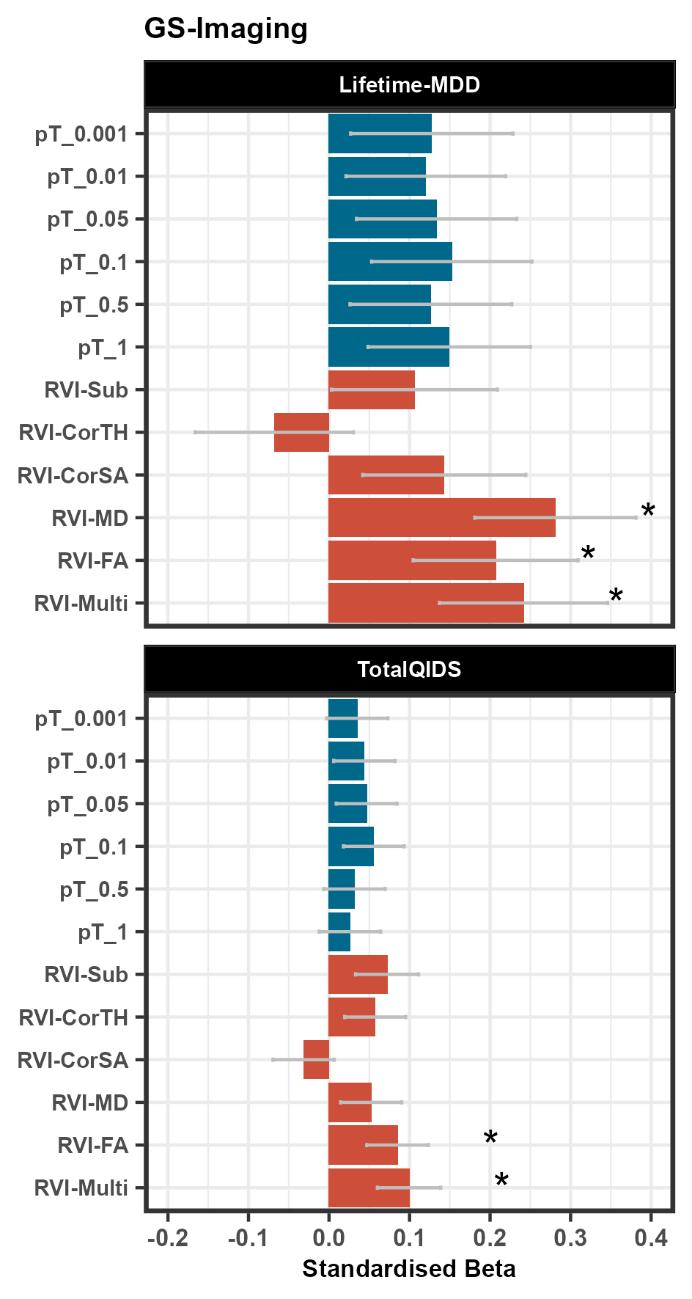
**

**Figure S3.** Association between MDD-RVIs/MDD-PRS (all thresholds) with Lifetime-MDD and TotalQIDS in GS-Imaging. The x-axis represents the standardised effect sizes and the y-axis represent the different RVI types and the MDD-PRS calculated at the different p-value thresholds.


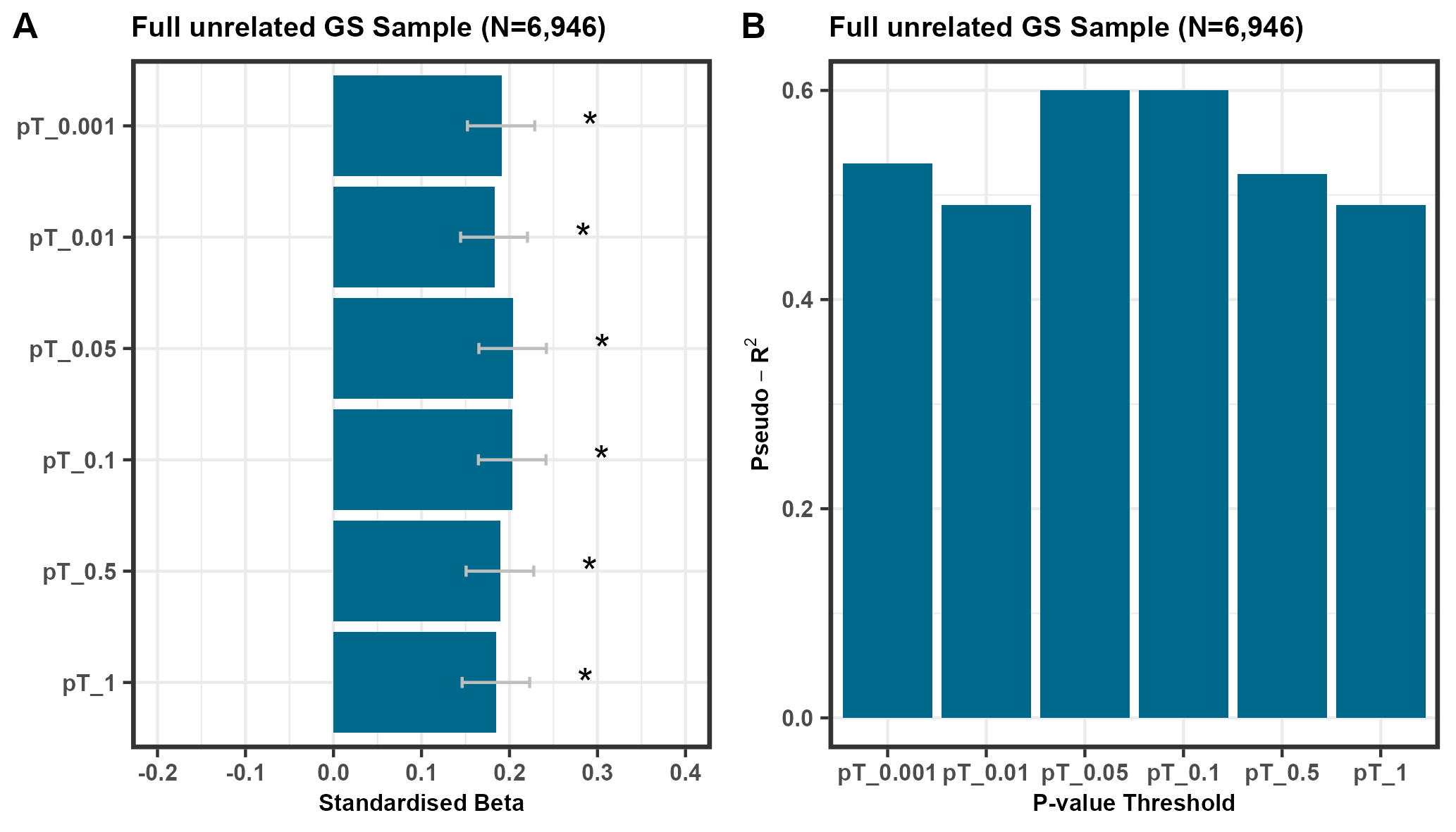


**Figure S4.** (A) Association between MDD-PRS and lifetime MDD diagnosis in the full unrelated GS sample (N=6,946) defined in Howard et al [9]. Individuals were defined as cases if they were given a MDD diagnosis using the Structured Clinical Interview for DSM disorders (975 cases and 5,975 controls). The x-axis represents the standardised effect sizes and the y-axis represent the different RVI types and the MDD-PRS calculated at the different p-value thresholds. (B) The change in McFadden Pseudo-R^2^ was calculated for each p-value threshold to assess the improvement in model fit upon adding MDD-PRS as a predictor, relative to the null model including only covariates.


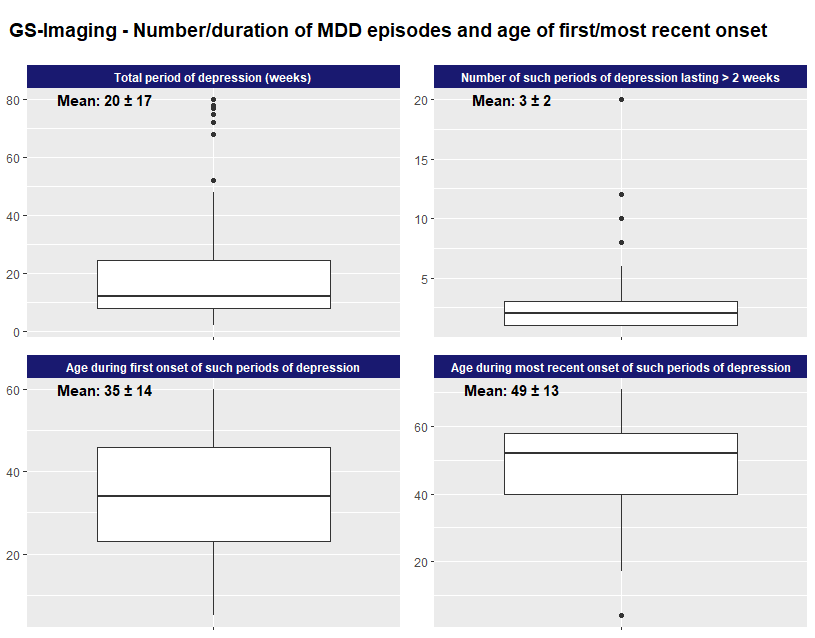


**Figure S5**. Additional information on number/duration of MDD episodes and age of first/most recent onset in GS-Imaging.

**Supplementary figures for ABCD**


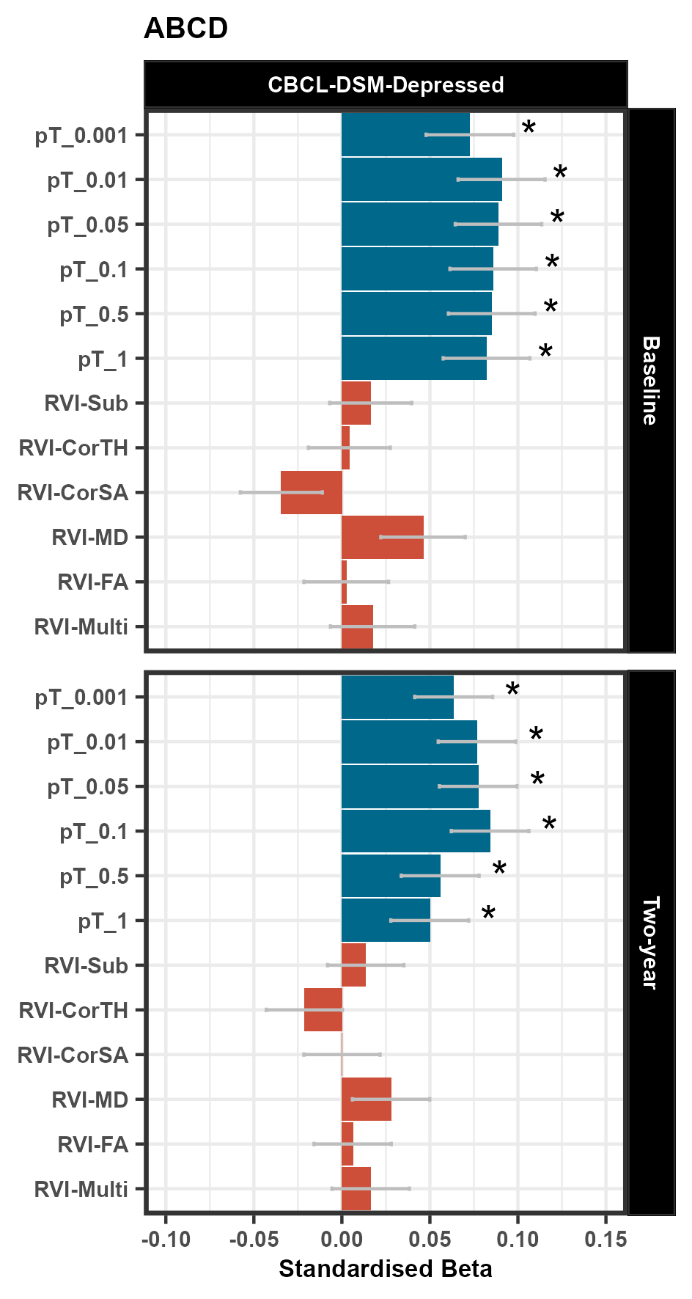


**Figure S6.** Association between MDD-RVIs/ MDD-PRS with CBCL-DSM-Depressed at baseline and two-year follow-up. The x-axis represents the standardised effect sizes and the y-axis represent the different RVI types and the MDD-PRS calculated at the different p-value thresholds.

**Supplementary Tables**

**ENIGMA data tables**

**Table S1**. MDD case-control effect sizes for subcortical volume obtained from Table 1 in Schmaal et al [10]. Covariates included age, sex, site and ICV.

| **Region** | **Cohen's d** |
| --- | --- |
| Accumbens | -0.019 |
| Amygdala | -0.060 |
| Caudate | -0.023 |
| Hippocampus | -0.144 |
| Pallidum | -0.001 |
| Putamen | 0.012 |
| Thalamus | -0.044 |

**Table S2.** MDD case-control effect sizes for cortical surface area and thickness obtained from Table S19 and Table 1 in Schmaal et al [11], respectively. Covariates included age, sex and site. The left and right hemispheres were averaged to calculate cortical-based RVIs in the current analysis.

| **Region** | **Cohen’s d –**  **Cortical SA** | **Cohen’s d –**  **Cortical Thickness** |
| --- | --- | --- |
| Left banks superior temporal sulcus | -0.024 | −0.058 |
| Left caudal anterior cingulate cortex | 0.021 | −0.042 |
| Left caudal middle frontal gyrus | 0.045 | −0.014 |
| Left cuneus | -0.007 | 0.05 |
| Left entorhinal cortex | 0.008 | −0.041 |
| Left frontal pole | -0.049 | −0.011 |
| Left fusiform gyrus | 0.012 | −0.117 |
| Left hemisphere total surface area | 0.026 | −0.057 |
| Left inferior parietal cortex | -0.005 | −0.063 |
| Left inferior temporal gyrus | 0.046 | −0.049 |
| Left insula | 0.014 | −0.111 |
| Left isthmus cingulate cortex | 0.063 | −0.104 |
| Left lateral occipital cortex | 0.016 | −0.023 |
| Left lateral orbitofrontal cortex | 0.041 | −0.046 |
| Left lingual gyrus | 0.028 | 0.01 |
| Left medial orbitofrontal cortex | 0.035 | −0.134 |
| Left middle temporal gyrus | -0.034 | −0.090 |
| Left paracentral lobule | 0.018 | −0.003 |
| Left parahippocampal gyrus | -0.017 | −0.072 |
| Left pars opercularis | -0.015 | −0.063 |
| Left pars orbitalis | -0.002 | −0.073 |
| Left pars triangularis | -0.063 | −0.054 |
| Left pericalcarine cortex | -0.008 | 0.09 |
| Left postcentral gyrus | 0.006 | 0.04 |
| Left posterior cingulate cortex | 0.006 | −0.099 |
| Left precentral gyrus | 0.034 | −0.020 |
| Left precuneus | 0.011 | −0.024 |
| Left rostral anterior cingulate cortex | 0.04 | −0.130 |
| Left rostral middle frontal gyrus | 0.025 | −0.037 |
| Left superior frontal gyrus | 0.052 | −0.066 |
| Left superior parietal cortex | 0.017 | −0.005 |
| Left superior temporal gyrus | 0.011 | 0.01 |
| Left supramarginal gyrus | 0.04 | −0.045 |
| Left temporal pole | 0.066 | 0.01 |
| Left transverse temporal gyrus | 0.013 | −0.035 |
| Right banks superior temporal sulcus | 0.01 | −0.074 |
| Right caudal anterior cingulate cortex | 0.011 | −0.080 |
| Right caudal middle frontal gyrus | 0.009 | 0.01 |
| Right cuneus | 0.058 | 0.05 |
| Right entorhinal cortex | 0.036 | −0.055 |
| Right frontal pole | 0.024 | −0.062 |
| Right fusiform gyrus | -0.034 | −0.116 |
| Right hemisphere total surface area | 0.007 | −0.049 |
| Right inferior parietal cortex | -0.024 | −0.041 |
| Right inferior temporal gyrus | 0.005 | −0.117 |
| Right insula | -0.004 | −0.115 |
| Right isthmus cingulate cortex | 0.04 | −0.071 |
| Right lateral occipital cortex | 0.067 | 0.01 |
| Right lateral orbitofrontal cortex | 0.026 | −0.120 |
| Right lingual gyrus | 0.016 | −0.012 |
| Right medial orbitofrontal cortex | 0.027 | −0.131 |
| Right middle temporal gyrus | 0.027 | −0.088 |
| Right paracentral lobule | 0.014 | −0.006 |
| Right parahippocampal gyrus | -0.04 | −0.061 |
| Right pars opercularis | -0.011 | −0.017 |
| Right pars orbitalis | 0.022 | −0.070 |
| Right pars triangularis | -0.012 | −0.031 |
| Right pericalcarine cortex | 0.007 | 0.08 |
| Right postcentral gyrus | 0.022 | 0.03 |
| Right posterior cingulate cortex | 0.016 | −0.093 |
| Right precentral gyrus | 0.009 | −0.022 |
| Right precuneus | 0.006 | 0.01 |
| Right rostral anterior cingulate cortex | 0.029 | −0.098 |
| Right rostral middle frontal gyrus | 0.054 | −0.038 |
| Right superior frontal gyrus | 0.028 | −0.078 |
| Right superior parietal cortex | -0.008 | 0.03 |
| Right superior temporal gyrus | 0.029 | −0.031 |
| Right supramarginal gyrus | 0.004 | −0.053 |
| Right temporal pole | 0.051 | 0.01 |
| Right transverse temporal gyrus | -0.021 | −0.051 |

**Table S3.** Tracts of interest listed in van Velzen et al [12]. Regions that match with ABCD and are used for the analyses are highlighted in yellow.

| **Abbreviation** | **Full tract name** |
| --- | --- |
| AverageFA | Full skeleton average FA |
| ACR (L+R) | Anterior corona radiata |
| ALIC (L+R) | Anterior limb of internal capsule |
| BCC | Body of corpus callosum |
| CC (BCC+GCC+SCC) | Corpus callosum |
| CGC (L+R) | Cingulum (cingulate gyrus) |
| CGH (L+R) | Cingulum (hippocampal portion) |
| CR (L+R) | Corona radiata |
| CST (L+R) | Corticospinal tract |
| EC (L+R) | External capsule |
| FX | Fornix |
| FXST (L+R) | Fornix (cres) / Stria terminalis |
| GCC | Genu of corpus callosum |
| IC (L+R) | Internal capsule |
| IFO (L+R) | Inferior fronto-occipital fasciculus |
| PCR (L+R) | Posterior corona radiata |
| PLIC (L+R) | Posterior limb of internal capsule |
| PTR (L+R) | Posterior thalamic radiation |
| RLIC (L+R) | Retrolenticular part of internal capsule |
| SCC | Splenium of corpus callosum |
| SCR (L+R) | Superior corona radiata |
| SFO (L+R) | Superior fronto-occipital fasciculus |
| SLF (L+R) | Superior longitudinal fasciculus |
| SS (L+R) | Sagittal stratum |
| UNC (L+R) | Uncinate fasciculus |

**Table S4.** Effect sizes for MD and FA obtained from Table S6 and Table S4 in van Velzen et al [12], respectively. Covariates included age, age^2^, sex, age*sex, age^2^*sex and site.

| **Region** | **Cohen's d – MD** | **Cohen's d – FA** |
| --- | --- | --- |
| AACR | 0.065 | -0.253 |
| ALIC | 0.124 | -0.232 |
| BCC | 0.192 | -0.243 |
| CC | 0.17 | -0.249 |
| CGC | 0.085 | -0.165 |
| CGH | 0.14 | -0.068 |
| CR | 0.12 | -0.25 |
| CST | 0.104 | -0.101 |
| EC | 0.148 | -0.16 |
| FX | 0.123 | -0.083 |
| FXST | 0.18 | -0.183 |
| GCC | 0.102 | -0.247 |
| IC | 0.104 | -0.229 |
| IFO | 0.093 | -0.121 |
| PCR | 0.143 | -0.202 |
| PLIC | 0.105 | -0.154 |
| PTR | 0.111 | -0.135 |
| RLIC | 0.075 | -0.151 |
| SCC | 0.143 | -0.13 |
| SCR | 0.192 | -0.197 |
| SFO | 0.164 | -0.232 |
| SLF | 0.126 | -0.169 |
| SS | 0.12 | -0.23 |
| UNC | 0.052 | -0.12 |
| Average | 0.176 | -0.258 |

**Table S5.** Standardised betas from linear regression analyses examining the association between FA values and severity of symptoms measured by the Beck Depression Inventory in adults only. This table was obtained from Table S92 in van Velzen et al [12].

| Region | Beta | SE | CI LB | CI UB | P-value | FDR P-value | I^2^ | N |
| --- | --- | --- | --- | --- | --- | --- | --- | --- |
| ACR | 0.000313 | 0.000187 | -0.000053 | 0.000679 | 0.093 | 0.883 | 34.109 | 477 |
| ALIC | 0.000088 | 0.000139 | -0.000184 | 0.000360 | 0.528 | 0.975 | 19.424 | 477 |
| BCC | 0.000020 | 0.000165 | -0.000304 | 0.000344 | 0.904 | 0.975 | 0.000 | 477 |
| CC | 0.000006 | 0.000123 | -0.000235 | 0.000246 | 0.963 | 0.975 | 0.276 | 477 |
| CGC | 0.000029 | 0.000261 | -0.000482 | 0.000539 | 0.913 | 0.975 | 41.845 | 477 |
| CGH | -0.000359 | 0.000326 | -0.000997 | 0.000279 | 0.269 | 0.975 | 50.839 | 477 |
| CR | 0.000165 | 0.000131 | -0.000091 | 0.000420 | 0.208 | 0.975 | 17.340 | 477 |
| CST | -0.000346 | 0.000502 | -0.001331 | 0.000638 | 0.491 | 0.975 | 81.332 | 477 |
| EC | 0.000018 | 0.000202 | -0.000378 | 0.000414 | 0.928 | 0.975 | 56.521 | 477 |
| FX | -0.000419 | 0.000259 | -0.000926 | 0.000089 | 0.106 | 0.883 | 0.000 | 477 |
| FXST | -0.000389 | 0.000214 | -0.000809 | 0.000031 | 0.069 | 0.883 | 31.235 | 477 |
| GCC | -0.000075 | 0.000236 | -0.000537 | 0.000388 | 0.752 | 0.975 | 47.584 | 477 |
| IC | -0.000019 | 0.000096 | -0.000206 | 0.000169 | 0.847 | 0.975 | 0.000 | 477 |
| IFO | -0.000330 | 0.000487 | -0.001285 | 0.000625 | 0.498 | 0.975 | 78.024 | 477 |
| PCR | 0.000054 | 0.000112 | -0.000167 | 0.000274 | 0.633 | 0.975 | 0.000 | 477 |
| PLIC | -0.000032 | 0.000124 | -0.000276 | 0.000211 | 0.794 | 0.975 | 0.000 | 477 |
| PTR | -0.000131 | 0.000133 | -0.000392 | 0.000130 | 0.326 | 0.975 | 0.000 | 477 |
| RLIC | -0.000129 | 0.000118 | -0.000361 | 0.000103 | 0.276 | 0.975 | 0.000 | 477 |
| SCC | 0.000055 | 0.000099 | -0.000139 | 0.000249 | 0.576 | 0.975 | 0.000 | 477 |
| SCR | 0.000208 | 0.000215 | -0.000214 | 0.000631 | 0.333 | 0.975 | 53.738 | 477 |
| SFO | 0.000122 | 0.000365 | -0.000593 | 0.000837 | 0.738 | 0.975 | 71.573 | 477 |
| SLF | 0.000045 | 0.000114 | -0.000178 | 0.000267 | 0.695 | 0.975 | 0.000 | 477 |
| SS | 0.000050 | 0.000119 | -0.000183 | 0.000282 | 0.677 | 0.975 | 0.000 | 477 |
| UNC | -0.000023 | 0.000232 | -0.000477 | 0.000431 | 0.921 | 0.975 | 5.682 | 477 |
| AverageFA | 0.000003 | 0.000078 | -0.000151 | 0.000156 | 0.975 | 0.975 | 5.459 | 477 |

**Table S6.** Standardised betas from linear regression analyses examining the association between MD values and severity of symptoms measured by the Beck Depression Inventory in adults only. This table was obtained from Table S94 in van Velzen et al [12].

| Region | Beta | SE | CI LB | CI UB | P-value | FDR P-value | I^2^ | N |
| --- | --- | --- | --- | --- | --- | --- | --- | --- |
| ACR | -0.00000001 | 0.00000031 | -0.00000061 | 0.00000059 | 0.975 | 0.975 | 61.795 | 477 |
| ALIC | -0.00000006 | 0.00000012 | -0.00000029 | 0.00000017 | 0.620 | 0.902 | 2.768 | 477 |
| BCC | 0.00000011 | 0.00000018 | -0.00000024 | 0.00000047 | 0.541 | 0.902 | 0.000 | 477 |
| CC | 0.00000005 | 0.00000015 | -0.00000025 | 0.00000035 | 0.745 | 0.902 | 1.767 | 477 |
| CGC | 0.00000016 | 0.00000015 | -0.00000014 | 0.00000046 | 0.297 | 0.902 | 9.121 | 477 |
| CGH | 0.00000025 | 0.00000020 | -0.00000014 | 0.00000064 | 0.207 | 0.902 | 15.588 | 477 |
| CR | 0.00000005 | 0.00000020 | -0.00000034 | 0.00000044 | 0.794 | 0.902 | 41.998 | 477 |
| CST | 0.00000013 | 0.00000024 | -0.00000034 | 0.00000061 | 0.586 | 0.902 | 41.516 | 477 |
| EC | 0.00000003 | 0.00000011 | -0.00000019 | 0.00000025 | 0.794 | 0.902 | 0.000 | 477 |
| FX | -0.00000023 | 0.00000112 | -0.00000243 | 0.00000197 | 0.838 | 0.908 | 11.894 | 477 |
| FXST | 0.00000017 | 0.00000013 | -0.00000009 | 0.00000043 | 0.191 | 0.902 | 14.567 | 477 |
| GCC | 0.00000003 | 0.00000017 | -0.00000031 | 0.00000037 | 0.872 | 0.908 | 0.000 | 477 |
| IC | -0.00000008 | 0.00000010 | -0.00000027 | 0.00000012 | 0.441 | 0.902 | 0.000 | 477 |
| IFO | 0.00000043 | 0.00000034 | -0.00000025 | 0.00000111 | 0.213 | 0.902 | 78.643 | 477 |
| PCR | 0.00000013 | 0.00000013 | -0.00000012 | 0.00000038 | 0.301 | 0.902 | 0.000 | 477 |
| PLIC | -0.00000004 | 0.00000013 | -0.00000028 | 0.00000021 | 0.761 | 0.902 | 0.000 | 477 |
| PTR | 0.00000016 | 0.00000016 | -0.00000015 | 0.00000047 | 0.316 | 0.902 | 11.768 | 477 |
| RLIC | -0.00000012 | 0.00000011 | -0.00000033 | 0.00000010 | 0.298 | 0.902 | 0.000 | 477 |
| SCC | -0.00000005 | 0.00000016 | -0.00000037 | 0.00000027 | 0.775 | 0.902 | 15.737 | 477 |
| SCR | 0.00000011 | 0.00000010 | -0.00000009 | 0.00000031 | 0.284 | 0.902 | 0.000 | 477 |
| SFO | 0.00000005 | 0.00000015 | -0.00000023 | 0.00000034 | 0.718 | 0.902 | 0.000 | 477 |
| SLF | 0.00000003 | 0.00000010 | -0.00000016 | 0.00000023 | 0.737 | 0.902 | 0.000 | 477 |
| SS | 0.00000009 | 0.00000016 | -0.00000023 | 0.00000040 | 0.585 | 0.902 | 20.731 | 477 |
| UNC | 0.00000022 | 0.00000024 | -0.00000025 | 0.00000070 | 0.353 | 0.902 | 0.000 | 477 |
| AverageMD | 0.00000005 | 0.00000009 | -0.00000013 | 0.00000024 | 0.562 | 0.902 | 0.000 | 477 |

**Supplementary tables for main analysis**

**Table S7.** Delta AIC values (e.g., “M2-M1” refers to the difference between M2 and M1 AIC values) for each model type (M1 to M5) when MDD-RVIs and MDD-PRS are used as predictors individually or in conjunction with each other. For model comparison, negative delta AIC values are indicative of better model fit. The results for both GS-Imaging and ABCD are reported.

| ***GS-Imaging*** | | | | | | | | | | | | | |
| --- | --- | --- | --- | --- | --- | --- | --- | --- | --- | --- | --- | --- | --- |
|  |  | **Lifetime-MDD** | | | | | | **TotalQIDS** | | | | | |
| **Model** | **Variables** | **RVI-Sub** | **RVI-CorTH** | **RVI-CorSA** | **RVI-MD** | **RVI-FA** | **RVI-Multi** | **RVI-Sub** | **RVI-CorTH** | **RVI-CorSA** | **RVI-MD** | **RVI-FA** | **RVI-Multi** |
| M1 | Covs (for RVI) | NIL | | | | | | NIL | | | | | |
| M2-M1 | RVI+Covs (for RVI) | -38.428 | -24.372 | -13.828 | -21.965 | -18.047 | -57.694 | -122.529 | -89.208 | -37.53 | -52.889 | -56.001 | -174.54 |
| M3 | Covs | NIL | | | | | | NIL | | | | | |
| M4-M3 | PRS+Covs | -0.363 | | | | | | -0.273 | | | | | |
| M5-M4 | PRS+RVI+Covs | -38.347 | -24.715 | -12.897 | -22.276 | -19.151 | -58.55 | -124.326 | -90.384 | -39.086 | -54.432 | -56.834 | -177.55 |
| ***ABCD*** | | | | | | | | | | | | | |
|  |  | **CBCL-DSM-Depressed (Baseline)** | | | | | | **CBCL-DSM-Depressed (Two-year)** | | | | | |
| **Model** | **Variables** | **RVI-Sub** | **RVI-CorTH** | **RVI-CorSA** | **RVI-MD** | **RVI-FA** | **RVI-Multi** | **RVI-Sub** | **RVI-CorTH** | **RVI-CorSA** | **RVI-MD** | **RVI-FA** | **RVI-Multi** |
| M1 | Covs (for RVI) | NIL | | | | | | NIL | | | | | |
| M2-M1 | RVI+Covs (for RVI) | 1.497 | 1.966 | -0.212 | -256.563 | -252.825 | -253.354 | 1.605 | 1.017 | 2 | -163.139 | -161.579 | -162.062 |
| M3 | Covs | NIL | | | | | | NIL | | | | | |
| M4-M3 | PRS+Covs | -11.385 | | | | | | -12.823 | | | | | |
| M5-M4 | PRS+RVI+Covs | 0.892 | 1.988 | 0.494 | -253.625 | -249.971 | -251.168 | 1.504 | 0.734 | 1.977 | -164.721 | -163.399 | -163.875 |

*Covs: Covariates; “Covs (for RVI)“ models do not include the 15 genetic principal components and genotype plate number that were included in the “Covs” models

**Table S8.** Association between baseline MDD-RVIs and MDD-PRS with the residualised CBCL-DSM-Depressed scores from baseline to two-year follow up for all RVI types and p-value thresholds.

| **Dependent variable** | **Predictor** | ***B*** | **SD** | **t-value** | **p-value** |
| --- | --- | --- | --- | --- | --- |
| CBCL-DSM-Depressed (residualised) | pT_0.001 | 0.022 | 0.028 | 0.784 | 0.433 |
|  | pT_0.01 | 0.034 | 0.028 | 1.201 | 0.230 |
|  | pT_0.05 | 0.026 | 0.028 | 0.914 | 0.361 |
|  | pT_0.1 | 0.030 | 0.028 | 1.087 | 0.277 |
|  | pT_0.5 | 0.004 | 0.028 | 0.146 | 0.884 |
|  | pT_1 | -0.001 | 0.028 | -0.046 | 0.963 |
|  | RVI-Sub | 0.009 | 0.026 | 0.331 | 0.741 |
|  | RVI-CorTH | -0.024 | 0.026 | -0.916 | 0.360 |
|  | RVI-CorSA | 0.022 | 0.026 | 0.842 | 0.400 |
|  | RVI-MD | -0.003 | 0.027 | -0.095 | 0.924 |
|  | RVI-FA | -0.007 | 0.027 | -0.248 | 0.804 |
|  | RVI-Multi | -0.008 | 0.026 | -0.284 | 0.777 |

**References**

1. The 1000 Genomes Project Consortium. A global reference for human genetic variation.*Nature* 2015; 526: 68-74.
2. Lam M, Awsasthi S, Watson HJ, Goldstein J, Panagiotaropoulou G, Trubetskoy V, et al. RICOPILI: Rapid Imputation for COnsortias PIpeLIne. *Bioinformatics* 2020; 36: 930–933.
3. Habota T, Sandu A, Waiter GD, McNeil CJ , Steele JD, Macfarlane JA, *et al*. Cohort profile for the STratifying resilience and depression longitudinally (STRADL) study: A depression-focused investigation of generation scotland, using detailed clinical, cognitive, and neuroimaging assessments.*Wellcome Open Research* 2019; 4:185.
4. Neilson E, Shen X, Cox SR, Clarke T, Wigmore EM, Gibson J, *et al*. (2019). Impact of polygenic risk for schizophrenia on cortical structure in UK biobank.*Biol Psychiatry* 2019; 86: 536-544.
5. Green C, Shen X, Stevenson AJ, Conole ELS, Harris MA, Barbu MC, et al. Structural brain correlates of serum and epigenetic markers of inflammation in major depressive disorder.*Brain, Behavior, and Immunity* 2021; 92: 39-48.
6. Stolicyn A, Harris MA, Shen X, Barbu MC, Adams MJ, Hawkins EL, *et al*. Automated classification of depression from structural brain measures across two independent community‐based cohorts.*Hum Brain Mapp* 2020; 41: 3922-3937.
7. Hagler DJ Jr, Hatton S, Cornejo MD, Makowski C, Fair DA, Dick AS, *et al*. Image processing and analysis methods for the Adolescent Brain Cognitive Development Study. *NeuroImage* 2019; 202: 116091.
8. Shen X, MacSweeney N, Chan SWY, Barbu MC, Adams MJ, Lawrie SM, *et al*. Brain structural associations with depression in a large early adolescent sample (the ABCD study®). *EClinicalMedicine* 2021; 42:101204.
9. Howard DM, Adams MJ, Clarke TK, Hafferty JD, Gibson J, Shirali M, *et al*. Genome-wide meta-analysis of depression identifies 102 independent variants and highlights the importance of the prefrontal brain regions. *Nat Neurosci* 2019; 22: 343-352.
10. Schmaal L, Veltman DJ, van Erp TG, Sämann PG, Frodl T, Jahanshad N, *et al*. Subcortical brain alterations in major depressive disorder: findings from the ENIGMA Major Depressive Disorder working group. *Mol Psychiatry* 2016; 21: 806-12.
11. Schmaal L, Hibar DP, Sämann PG, Hall GB, Baune BT, Jahanshad N, *et al*. Cortical abnormalities in adults and adolescents with major depression based on brain scans from 20 cohorts worldwide in the ENIGMA Major Depressive Disorder Working Group. *Mol Psychiatry* 2017; 22: 900-909.
12. van Velzen LS, Kelly S, Isaev D, Aleman A, Aftanas LI, Bauer J, *et al*. White matter disturbances in major depressive disorder: a coordinated analysis across 20 international cohorts in the ENIGMA MDD working group. *Mol Psychiatry* 2020; 25: 1511-1525.
